# Multistate model of the patient flow process in the pediatric emergency department

**DOI:** 10.1371/journal.pone.0219514

**Published:** 2019-07-10

**Authors:** Anqi Liu, David M. Kline, Guy N. Brock, Bema K. Bonsu

**Affiliations:** 1 Department of Biomedical Informatics, College of Medicine, The Ohio State University, Columbus, OH, United States of America; 2 Division of Biostatistics, College of Public Health, The Ohio State University, Columbus, OH, United States of America; 3 Center for Biostatistics, Wexner Medical Center, The Ohio State University, Columbus, OH, United States of America; 4 Department of Pediatrics, Nationwide Children's Hospital (NCH) and The Ohio State University, Columbus, OH, United States of America; 5 Biostatistics Resource at NCH (BRANCH), Nationwide Children's Hospital and The Ohio State University, Columbus, OH, United States of America; 6 Division of Emergency Medicine, Nationwide Children's Hospital, Columbus, OH, United States of America; Duke University School of Medicine, UNITED STATES

## Abstract

**Objectives:**

The main purpose of this paper was to model the process by which patients enter the ED, are seen by physicians, and discharged from the Emergency Department at Nationwide Children’s Hospital, as well as identify modifiable factors that are associated with ED lengths of stay through use of multistate modeling.

**Methods:**

In this study, 75,591 patients admitted to the ED from March 1^st^, 2016 to February 28^th^, 2017 were analyzed using a multistate model of the ED process. Cox proportional hazards models with transition-specific covariates were used to model each transition in the multistate model and the Aalen-Johansen estimator was used to obtain transition probabilities and state occupation probabilities in the ED process.

**Results:**

Acuity level, season, time of day and number of ED physicians had significant and varying associations with the six transitions in the multistate model. Race and ethnicity were significantly associated with transition to left without being seen, but not with the other transitions. Conversely, age and gender were significantly associated with registration to room and subsequent transitions in the model, though the magnitude of association was not strong.

**Conclusions:**

The multistate model presented in this paper decomposes the overall ED length of stay into constituent transitions for modeling covariate-specific effects on each transition. This allows physicians to understand the ED process and identify which potentially modifiable covariates would have the greatest impact on reducing the waiting times in each state in the model.

## Introduction

Accurate prediction and reduction of emergency department (ED) waiting times (typically defined as time to first physician contact) is of critical importance to emergency patients, as recent data has shown increasing waiting times and visit volumes in both the United States and Canada [1]. Prolonged ED wait times can result in numerous problems. These include reduced quality of clinical care, including an increase in clinical errors and complications [2] all of which results in declining overall patient satisfaction [3]. Disease progression can be non-linear in some children with precipitous and calamitous deterioration after periods of apparent stability. Prompt evaluation and treatment is necessary to avoid poor outcomes. Long ED wait times can also result in economic losses for families seeking care and for healthcare facilities. Time spent waiting hurts work productivity for families and fosters patient elopements, causing hospitals to squander achievable revenue by losing patients who could have been seen [4]. To combat these issues, some hospitals have developed mobile applications which display waiting times on the screens of users. Thus, patients can choose the hospital which fits their situation the best based on both distances from their home to the hospitals and hospital waiting times. However, posted ED waiting times in hospitals are not always accurate [5].

There have been many studies which developed models of ED waiting times and relevant predictors [6–10]. However, previous research has focused on modeling either the time to first physician contact or the entire ED length of stay and thus cannot provide insights concerning different periods of the ED process. For example, how do patient, environmental, seasonal, process flow, personnel, platform, and system-level factors interact to influence various ED intervals such as the time from first physician contact to disposition and subsequently to discharge? Further, patients who leave the ED without being seen pose a challenge for standard regression modeling approaches. Multistate models, which model the transitions of subjects between a finite number of states, pose a natural solution for both of these issues. The time between each successive event in the ED can be modeled separately and then synthesized into an overall transition probability model, and the subjects who leave without being seen can be right-censored at their last observed time in the ED. While multistate models have been widely applied in the biomedical literature [11–13], to our knowledge no one has previously used a multistate model for the ED process. Consequently, in this work we develop and apply a multistate model to ED waiting times and investigate factors associated with transitions between each state in the process.

In this work we use multistate models to model the process by which patients present to and are ultimately discharged from the Pediatric Emergency Department at Nationwide Children’s Hospital, including transitions between initial registration, exam room, initial physician contact, disposition, and discharge from the ED. Transitions between each state are modeled using patient, seasonal, time-of-day, and system-level variables to identify potentially modifiable factors that could improve patient flow and reduce waiting times.

## Materials and methods

### Study setting and population

Data were obtained from all Emergency Department (ED) visits at the Nationwide Children’s Hospital (NCH) from March 1^st^, 2016, to February 28^th^, 2017. Only patients who walked into the ED were included in the dataset. Patients from the Trauma Center and who arrived via emergency transportation were excluded as they followed a different process. Patients with transition times which we considered inaccurate or invalid (e.g., discharge time occurred prior to time first seen by physician) were also removed. Institutional review board (IRB) approval was obtained from both NCH and The Ohio State University (IRB17-00792). Since the study is retrospective the IRB waived the requirement for informed consent which allowed data with identifiers to be accessed by the investigators. Fully de-identified data are available as supplementary material (S1 Data), along with a description of the variables (S2 Data).

### Outcomes and covariates

The outcomes are time to each successive state in the ED patient flow with the unit of time as hours. The covariates included age, gender, race, ethnicity, acuity level (as measured or by the Emergency Severity Index (ESI)), season, time period of the day at registration and the number of physicians on staff at time of registration. Acuity was modeled as a time-varying covariate, with an initial level recorded at registration which could be updated at the time of triage. This incorporates the situation where staff update the acuity level in consideration of some newly found symptoms and vital signs after initial registration.

### Data analysis

**Fig 1:**
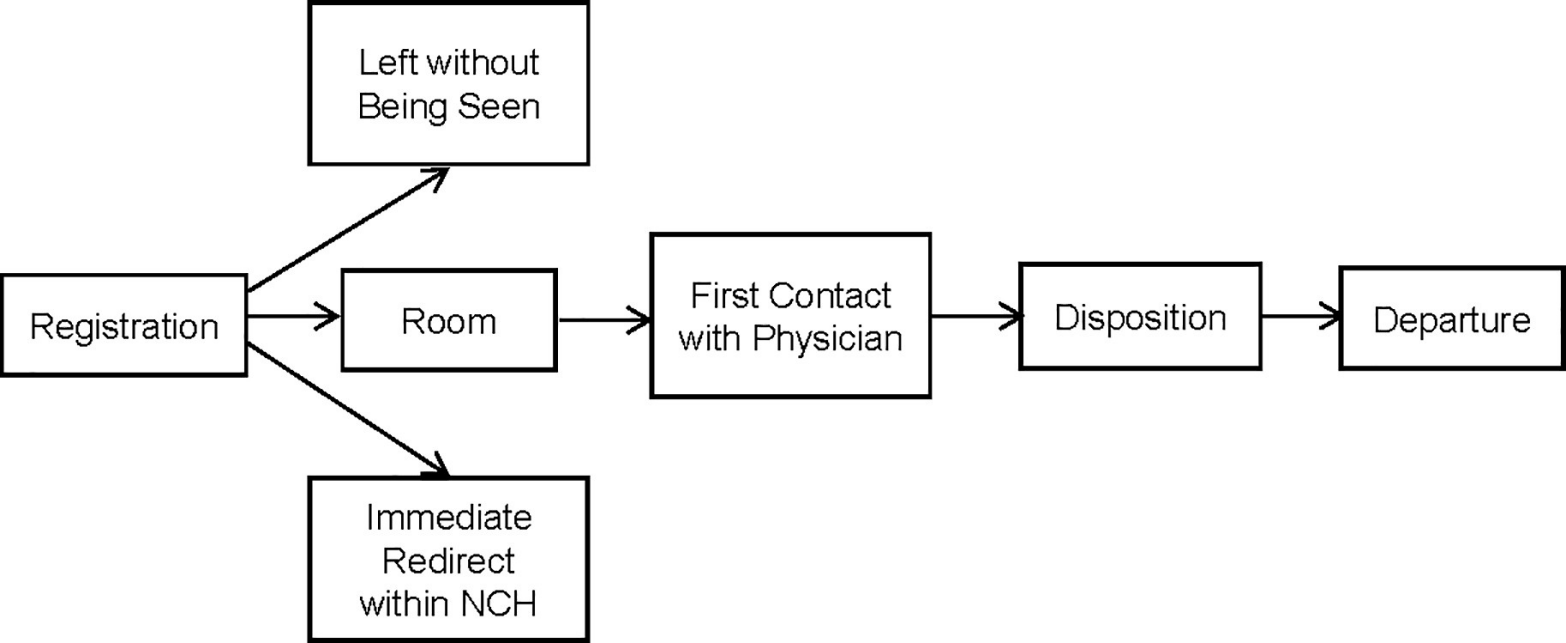
Multistate model of patient flow in the Emergency Department at Nationwide Children’s Hospital (NCH).

We utilized multistate models to analyze transition times between successive states in the ED process at Nationwide Children’s Hospital (NCH), as illustrated in Fig 1. The primary aim of the current study is to estimate state occupation probabilities and transition probabilities between the states in the ED process and to identify the relevant factors that are associated with increased or reduced waiting times for each state in the model.

In the multistate model, all patients start from registration and are either seen by a physician and subsequently discharged, leave without being seen, or are immediately redirected within NCH. Therefore, the initial state is Registration (state 1) and the terminal states are Left (state 2), Redirect (state 3), and Departure (state 7). Intermediate states consist of Exam Room (state 4), First Contact with Physicians (state 5) and Disposition (state 6). The total number of transitions between states is six. The R package *mstate* was used to estimate state occupation probabilities and transition probabilities for the multistate model of the ED process [14]. Time zero was set as when patients registered and times to each state refer to the time since patient registration. Relevant covariates in each transition were separately modeled using the Cox proportional hazards model with possibly time-varying covariates (e.g., acuity level). The proportional hazards assumption was tested using a global likelihood ratio test and visually inspected using log-minus-log survival plots for categorical covariates and scaled Schoenfeld residuals for continuous covariates [15]. Transition probabilities and state occupation probabilities were calculated using the Aalen-Johansen estimator [16]. All code to reproduce the results presented in this paper are available as supplementary material (S1 Code).

## Results

There were 78,123 people who walked into the ED included in the dataset. After removing subjects with missing or invalid transition times 75,591 subjects remained. Of these, 71,378 (94.4%) were discharged after being seen by a physician within the ED, 3,471 (4.6%) left without being seen, and 742 (1%) were immediate redirects. The mean waiting time for each transition state was 1.06 hours from registration to room, 0.5 hours from room to first contact with a physician, 1.4 hours from first contact to disposition, and 1.2 hours from disposition to departure. Table 1 displays demographic characteristics including age, gender, ethnicity and race, as well as frequency distributions of acuity levels, seasons, time of the day and the number of ED physicians. The study subjects in the population were more commonly male (51.5%), mostly not Hispanic or Latino (91.1%), white (48.0%), and had a median age of 5 years old (inter-quartile range (IQR) 2 to 12). Fewer subjects entered the ED in the summer (21.6%) compared to winter (26.9%), spring (25.7%), and fall (25.8%). The median number of ED physicians on staff was 7 with an IQR of 5 to 8, though this varied widely with time of day.

**Table 1:** Demographics and clinical characteristics.

Variable	Level	Value
Total		75,591
Age (median (IQR^1^))		5 (2 to 12)
Gender (%)	Male	38,927 (51.5)
	Female	36,664 (48.5)
Ethnicity (%)	Not Hispanic or Latino	68,922 (91.2)
	Hispanic or Latino	5,194 (6.9)
	Other	1,005 (1.3)
	Unknown	470 (0.6)
Race (%)	White	36,321 (48.0)
	Black or African American	26,908 (35.6)
	Asian	1,987 (2.6)
	Other	6,216 (8.2)
	Unknown	4,159 (5.5)
Acuity (ESI) (%)	1	100 (0.1)
	2	13,124 (17.4)
	3	24,106 (31.9)
	4	24,484 (32.4)
	5	13,777 (18.2)
Season (%)	Spring	19,401 (25.7)
	Summer	16,351 (21.6)
	Fall	19,486 (25.8)
	Winter	20,353 (26.9)
Time of day (%)	0:00–4:00	7,263 (9.6)
	4:00–8:00	4,192 (5.5)
	8:00–12:00	13,555 (17.9)
	12:00–16:00	16,333 (21.6)
	16:00–20:00	17,251 (22.8)
	20:00–24:00	16,997 (22.5)
The number of ED physicians (median (IQR^1^))		7 (5 to 8)

^1^IQR = inter-quartile range (25^th^ and 75^th^ percentiles)

Table 2 displays hazard ratios for the covariates for the two initial transitions of registration to room and registration to left without being seen. Age, gender, acuity level, season, time of day, and number of ED physicians were all statistically significant for the registration to room transition. However, the magnitude of association was relatively minor for age and gender and primarily driven by the large sample size. In contrast, higher ESI levels clearly had reduced registration to room transition rates compared to more emergent cases, as expected. Time of day and season also exhibited strong associations with registration to room times. For registration to left without being seen, again acuity level, season, and time of day were significantly associated. However in contrast to registration to room transitions, there was a significant association with race and ethnicity but not with age and gender. In this case, Asians and Hispanics had reduced rates of leaving without being seen compared to Whites, while Blacks had slightly higher rates.

**Table 2:** Cox models for registration to room and registration to left without being seen.

	Registration-Room			Registration-Left wo/Being Seen		
	HR	95% CI	P-value	HR	95% CI	P-value
Age	1.004	1.003, 1.005	<0.001	0.998	0.991, 1.004	0.45
Gender						
Male	Reference			Reference		
Female	0.963	0.949, 0.977	<0.001	1.002	0.937, 1.071	0.962
Ethnicity						
Not Hispanic	Reference			Reference		
Hispanic	1.029	0.994, 1.066	0.108	0.507	0.425, 0.604	<0.001
Other	0.97	0.908, 1.036	0.363	0.941	0.708, 1.249	0.672
Unknown	0.95	0.861, 1.047	0.299	1.769	1.285, 2.433	<0.001
Race						
White	Reference			Reference		
Black	0.999	0.983, 1.016	0.94	1.13	1.047, 1.219	0.002
Asian	1.029	0.982, 1.078	0.228	0.434	0.332, 0.567	<0.001
Other	0.99	0.962, 1.018	0.472	1.142	1.009, 1.293	0.036
Unknown	0.957	0.919, 0.997	0.035	1.151	0.973, 1.361	0.1
Acuity Level (ESI)						
1	Reference			Reference^1^		
2	0.224	0.182, 0.276	<0.001			
3	0.09	0.073, 0.11	<0.001	1.716	1.214, 2.427	0.002
4	0.075	0.061, 0.092	<0.001	3.082	2.188, 4.343	<0.001
5	0.069	0.056, 0.085	<0.001	3.702	2.62, 5.231	<0.001
Season						
Spring	Reference			Reference		
Summer	1.426	1.395, 1.458	<0.001	1.223	1.076, 1.39	0.002
Fall	0.61	0.598, 0.623	<0.001	0.79	0.718, 0.869	<0.001
Winter	0.58	0.568, 0.592	<0.001	0.715	0.65, 0.787	<0.001
Time of the day						
0:00–4:00	Reference			Reference		
4:00–8:00	2.193	2.094, 2.296	<0.001	1.588	1.296, 1.946	<0.001
8:00–12:00	3.056	2.959, 3.157	<0.001	1.204	1.015, 1.427	0.033
12:00–16:00	1.784	1.731, 1.838	<0.001	0.965	0.861, 1.082	0.546
16:00–20:00	1.874	1.813, 1.936	<0.001	1.085	0.961, 1.225	0.187
20:00–24:00	1.549	1.497, 1.602	<0.001	0.996	0.893, 1.11	0.937
Number of ED physicians	0.918	0.911, 0.925	<0.001	0.952	0.925, 0.98	0.001

^1^ Since no patients with ESI level 1 left without being seen, ESI levels 1 and 2 were merged to form a baseline group in this case.

**Fig 2:**
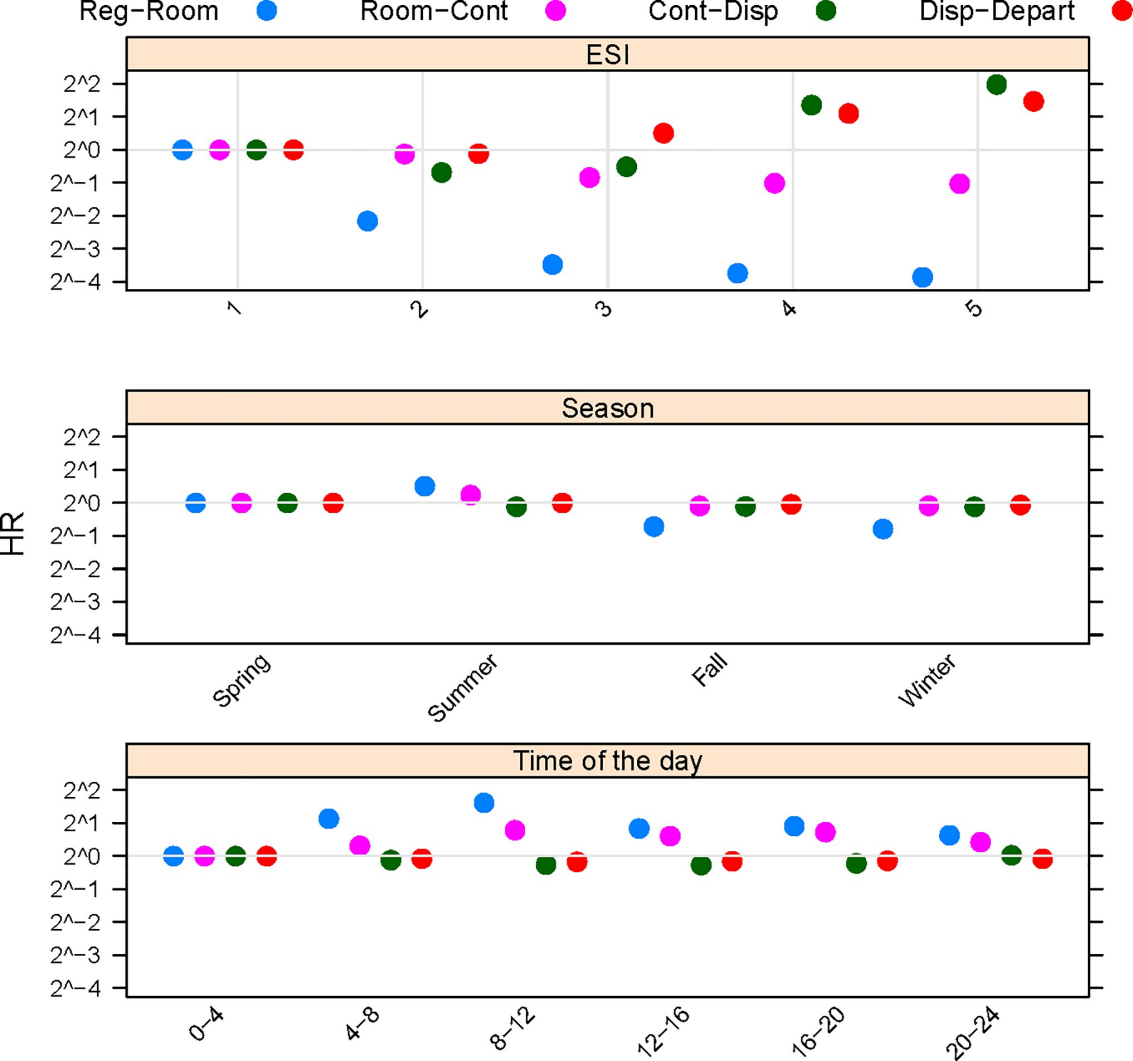
Forest plot of hazard ratios for ESI (acuity), season, and time of day (in hours). Hazard ratios are for transitions from Registration to Room (Reg-Room), Room to First Physician Contact (Room-Cont), First Physician Contact to Disposition (Cont-Disp), and Disposition to Departure (Disp-Depart).

Hazard ratios for all transitions in the multistate model are given in supplementary S1 Table, and are displayed graphically for registration to room and subsequent transitions for acuity level, season, and time of day in Fig 2. Fig 2 demonstrates the importance of modeling each transition in the multistate model, as the relative transition rates can depend on the transition in the ED model. For example, lower acuity (ESI 4–5) patients had much slower registration to room times (blue dots, Fig 2) and faster first physician contact to disposition and disposition to departure times (green and red dots, Fig 2). Another variable which had a varying association with transition times was the number of ED physicians. Somewhat counter-intuitively, higher number of ED physicians was associated with a reduced rate of registration to room times (HR = 0.918, Table 2) but also a reduced rate of leaving without being seen (HR = 0.925). However, increased number of ED physicians was associated with an increased rate first physician to disposition (HR = 1.019, S1 Table), which is more congruent with expectations. Number of ED physicians was not associated with room to first physician contact or disposition to departure transitions (S1 Table). In each of these cases, a model based on just time to first physician contact or total length of ED stay would miss important information.

**Fig 3:**
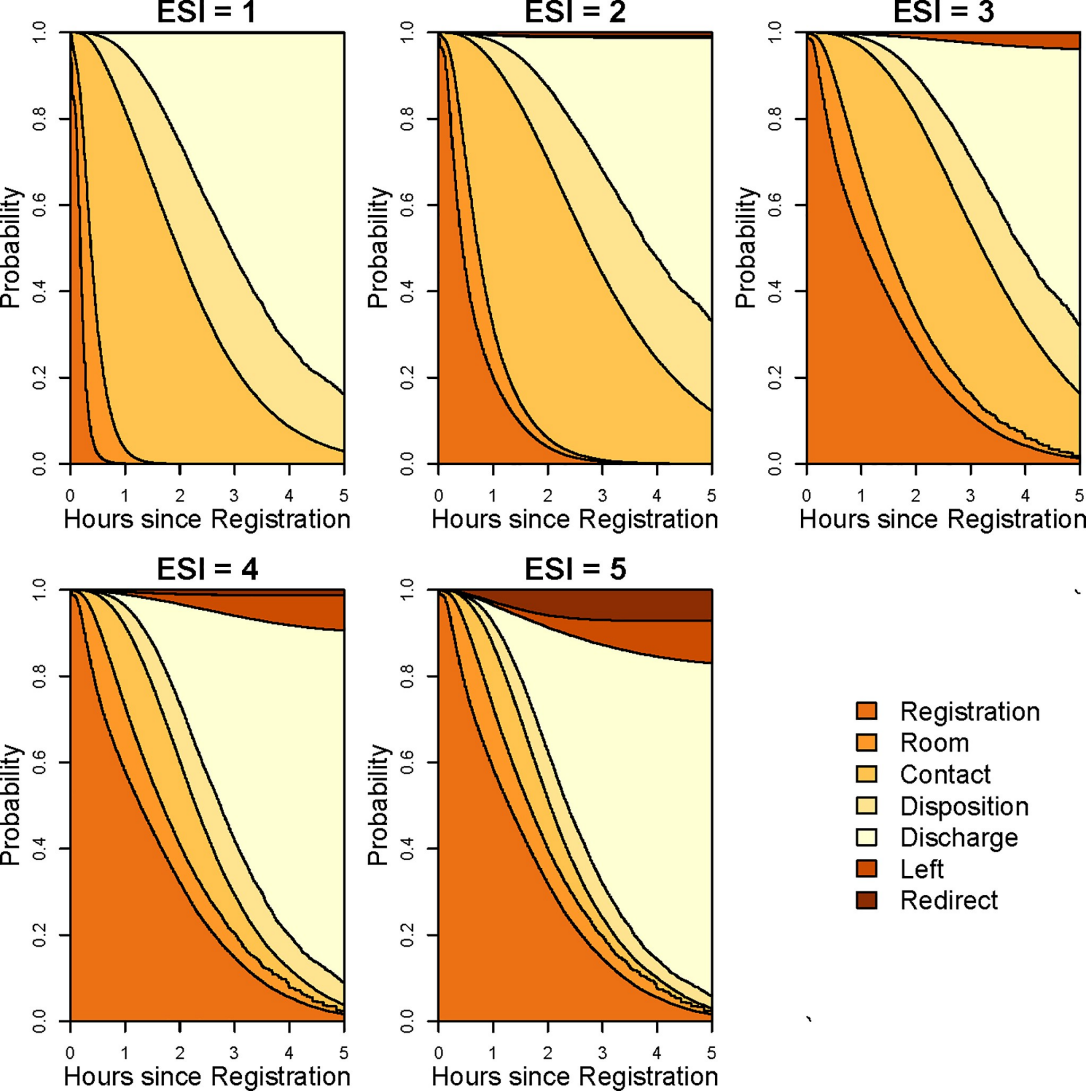
Stacked transition probabilities of the ED multistate model for different acuity (ESI) levels. Transition probabilities are conditional on age = 1 year, gender = male, ethnicity = not Hispanic or Latino, race = white, time of the day = 16:00–20:00, season = winter, and number of ED physicians = 8.

To better display the association between acuity level and waiting times, Fig 3 shows the stacked transition probabilities in the ED multistate model for different acuity levels. Predictions were obtained conditional on covariates, with the values of each covariate set to the most common value (age = 1 year, gender = male, ethnicity = not Hispanic or Latino, race = White, time of the day = 4:00–8:00 pm, season = winter, and number of ED physicians = 8). The vertical distance between two contiguous curves reflects the probability of being in the corresponding state at the given time, i.e. the state occupation probability. Patients with lower ESI and higher acuity had much faster admission to a room and first contact with a physician, whereas patients with higher ESI and lower acuity had faster transitions after first physician contact. These differences are further reflected in supplementary S1 Fig, where the expected transition time between registration and room is much faster for lower ESI (ESI 1 = 11.5 minutes, ESI 2 = 38.5 minutes, ESI 3 = 85.9 minutes, ESI 4 = 95.4 minutes, ESI 5 = 95.6 minutes) while the expected transition time between first physician contact and disposition (104.4, 131.7, 101.0, 31.5, and 19.7 minutes for ESI levels 1–5, respectively) and disposition and departure are much quicker for higher ESI.

**Fig 4:**
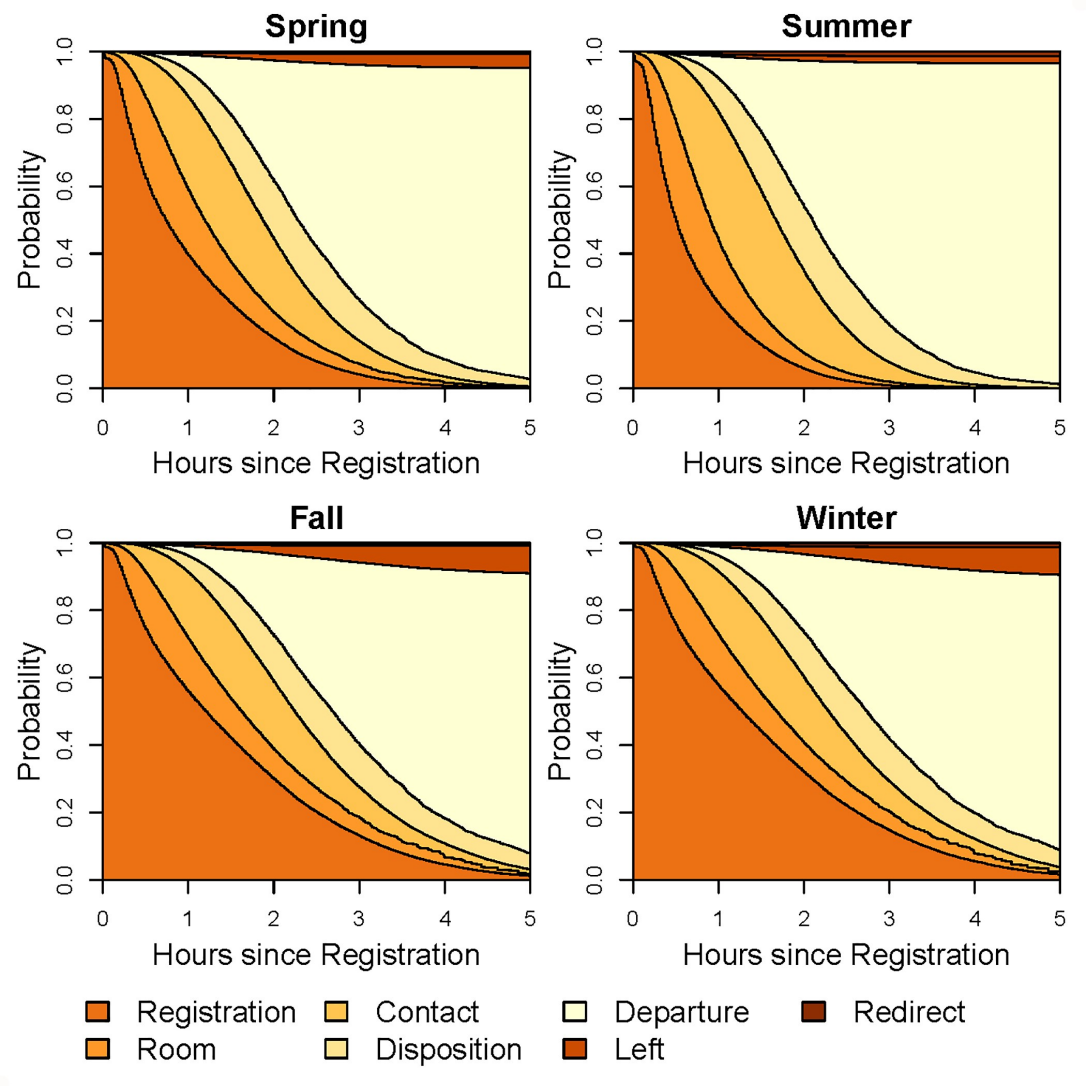
Stacked transition probabilities of the ED multistate model for different seasons. Transition probabilities are conditional on age = 1 year, gender = male, ethnicity = not Hispanic or Latino, race = white, time of the day = 16:00–20:00, ESI = 4, and number of ED physicians = 8.

**Fig 5:**
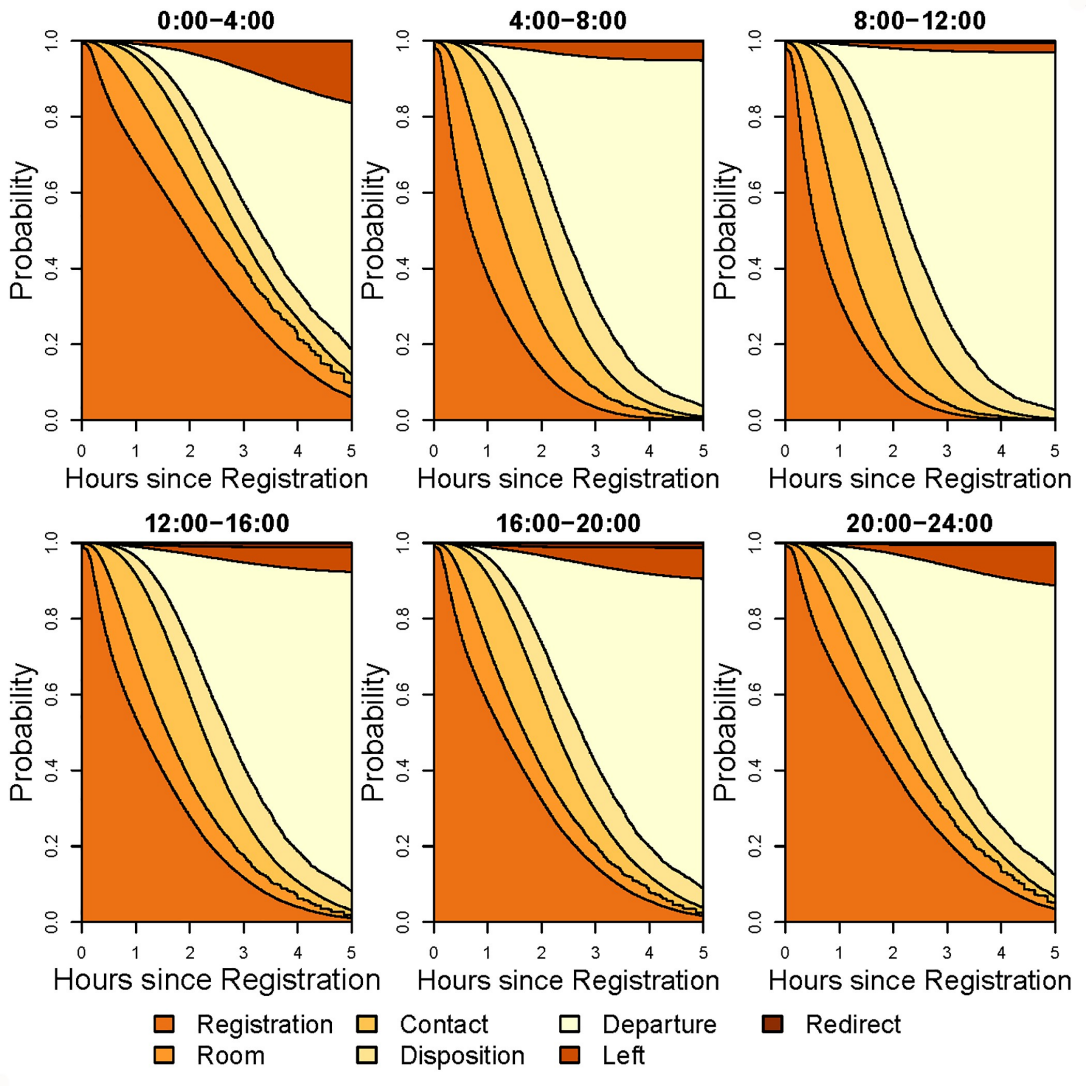
Stacked transition probabilities of the ED multistate model for different times of the day. Transition probabilities are conditional on age = 1 year, gender = male, ethnicity = not Hispanic or Latino, race = white, ESI = 4, season = winter. The number of ED physicians for each time of day was set to the median value for that period, as follows: 0:00–4:00 = 7, 4:00–8:00 = 3, 8:00–12:00 = 5, 12:00–16:00 = 6, 16:00–20:00 = 8, 20:00–24:00 = 8.

Figs 4 and 5 show stacked transition probabilities and supplementary S2 Fig and S3 Fig display expected lengths of stay (ELOS) stratified by season and time of day, respectively. For time of day, the number of physicians was set to the median value for that period to better reflect actuality. Patients seen during the winter and fall had slower transition times from registration to room and higher probability of patients leaving without being seen compared to the spring and summer. In contrast, patients seen during the summer had longer waiting times between first physician contact and disposition compared to the other seasons. In Fig 5, patients seen between midnight and 4:00 am had the slowest Registration to Room transition times and also the highest probability of patients leaving without being seen, followed by the 8:00 pm to midnight period. The fastest registration to room times occurred during the 4:00 am to noon period. Stacked transition probability plots and ELOS plots for race, ethnicity, and gender are given supplementary S4–S9 Figs. The most salient feature of these plots is the difference in probability of leaving without being seen for race (S4 Fig) and ethnicity (S6 Fig).

While several of the covariates in the Cox models for the transition times violated the assumption of proportional hazards based on the likelihood ratio test, visual inspection of log-minus-log survival plots did not reveal substantial violations in the majority of cases. A possible exception was ESI level, which had crossing cumulative hazards for several transitions (supplemental S10 Fig). For example, high acuity patients (ESI levels 1–3) initially transition much more quickly from registration to room than low acuity (ESI levels 4–5) patients. However, around 20–30 minutes rates for ESI level 4–5 patients accelerate and catch the rate for ESI level 3 patients. This indicates that after 30 minutes of waiting, a lower acuity (ESI 4–5) patient will be seen as quickly as a patient with an ESI of 3.

## Discussion

In this paper, the patient flow process in a pediatric emergency department was analyzed using multistate time-to-event models. The ED length of stay was separated into different phases including time from registration to exam room, exam room to first contact by a physician, first contact to disposition, and disposition to discharge. Transitions for registration to left without being seen and registration to immediate transfer within NCH were also included. The multistate model was used to assess how patient, institutional, seasonal and time of day factors influenced each transition. Our approach has several advantages over previous studies which modeled ED waiting times, in that a) it allows for different effects of covariates on each transition in the model and b) it naturally accounts for individuals who left without being seen. The multistate model then integrates the separate models for each transition to provide estimates of transition probabilities and state occupation probabilities. Allowing for transition-specific effects is important because many of the covariates had an effect which depended on the transition, most notably acuity level where lower acuity was associated with longer registration to exam room times but shorter first contact to disposition times. However, both season and time-of-day also had varying effects on the different transitions which relays important information which intuits with what physicians observe in practice. For example, time from registration to first contact was shorter in the summer compared to the fall and winter because overall patient volume was lower. This is likely attributable to spikes in seasonal illnesses like cold and flu which occur during the fall and winter months. However, time from first contact to disposition was longer in the summer compared to those two seasons. This could be partly attributable to a greater percentage of accidents presenting to the ED during the summer months, due to heightened outdoor activity during the warmer weather. Separately assessing each of these transitions provides a more complete picture of the ED flow process and allows hospital personnel to make staffing considerations which take into account these differences. For example, the number of ED physicians at different time periods can be adjusted or staff increased in the fall and winter in an effort to reduce ED waiting times.

Regression modeling of ED waiting times has several purposes, including predicting ED time to first physician contact or overall LOS [7, 8], identifying relevant factors which are potentially modifiable [4], developing a forecasting model to simulate effects of interventions [17, 18], and for evaluating an actual intervention based on observational data [19–21]. While our presentation focused on the first two of these aspects, the multistate model developed in this work is amenable to the latter two as well. Our conditional transition probability estimates from the multistate model can form the basis of a discrete event simulation model to forecast how changes in staffing and other hospital-level factors would affect transition probabilities between the states in the model [18]. The flow into the model could by varied to account for spikes and dips in patient influx and to evaluate limitations on the system. The multistate model could also be adapted to assess the effect of applying different strategies in the ED by using causal inference approaches [22]. Since a direct comparison of transition times in the ED model between two strategies ignores possible differences in case mix and other factors influencing ED length of stay, a causal inference approach is needed to account for the observational nature of the study and adjust for potential covariate differences. The end-goal is for the comparison to become more in line with a randomized trial. Examples of causal inference approaches with multistate models include artificially manipulating transition intensities, inverse probability weighting and G-computation [22]. Finally, the multistate model could be used in conjunction with a process control chart to assess ED quality control by maintaining expected or median waiting times in each state (e.g., on a monthly basis) within an acceptable range. Marginalized estimates which integrate over other covariates in the model, e.g. by using G-computation, would account for month-to-month fluctuations in patient mix and volume and be a better quality indicator compared to unadjusted ED time to first physician contact or overall LOS.

Previous investigations have found numerous predictors associated with ED waiting times [7, 8]. As reflected in our study, a crucial factor which can impact ED waiting times is triage. The primary goal of triage in the emergency department is to facilitate the treatment prioritization of patients based on the urgency of patients’ symptoms and vital signs [23]. Acuity level is an important index used in triage where a lower acuity level equates to less severe symptoms and vital signs. Many experts have studied the relationship between triage and ED waiting times [24]. Newton et al. showed that acuity level in triage was an important predictor of ED waiting time [10]. Also, Choi, Wong and Lau carried out a trial related to triage in the emergency department of a hospital and the mean waiting time in the ED was decreased by 38%, which meant that the triage intervention had a great effect in reducing ED waiting times [25]. Bruijns, Wallis and Burch reached the same conclusion after introducing triage [26]. Besides acuity level, researchers also identified additional relevant covariates for ED waiting times including race and ethnicity [6, 27] and the number of attending physicians [9]. In our paper, we discussed in detail the relationships between relevant covariates and different phases of the ED process. Race and ethnicity were found to be associated with the probability of leaving without being seen, though not with the other transitions in the model. In contrast, age and gender were not significantly associated with the left without being seen transition, but were significantly associated (though not strong in magnitude) with the other transitions in the model. Acuity level, season, and time of the day were all relevant factors for each transition.

One somewhat perplexing result is that increased number of ED physicians was associated with increased registration-to-room times, counter to expectation. A possible explanation is that a greater number of physicians are on staff during the busiest periods of the day, and while time of day is included in the model there is likely residual information that is captured in physician counts driving the association. Another explanation is that we could not include information on nurse staffing in the model, and this is arguably more relevant for registration to room times. A backlog of unfilled physician orders occurring during intervals of relative nurse paucity may result in re-allocation of nursing resources away from initial assessment/triage to bedside functions, prolonging registration-to-room times. Once roomed, however, it has been our observation that patients are less likely to elope untreated provided contact with a physician is timely, which is more likely on days with high physician coverage hours. In support of this, we observed that higher number of ED physicians was associated with decreased probability of leaving without being seen and increased rates of first physician contact to disposition.

In addition to reducing overall ED waiting times, an important outcome in evaluating pediatric EDs is the percentage of patients who left without being seen [4]. Not properly accounting for individuals who left without being seen could bias estimates of time to first physician contact and ED LOS. In our model included this as an additional transition within our model, highlighting the flexibility of the multistate modeling approach. This transition time is likely over-estimated in our model, as standard procedure is to call a patient in the lobby to the front desk for any intervention (vitals, nurse reassessment, rooming, X-rays, etc.) three times at intervals of 15 minutes apart before recording the patient as having left without being seen. Hence the patient has likely left prior to the time recorded in the system, though knowing exactly when is not possible currently.

One aspect of ED patient flow not directly investigated in this study is the issue of potential boarding delays resulting from admitted patients being held in ED until an inpatient bed is prepared [28–31]. This delay in transfer from the ED to the inpatient ward can have a strong impact on time to physician contact and length of stay and door to physician contact [29, 30]. While directly modeling inpatient boarding delays is complex, by breaking down the total ED length of stay into constituent parts we see potential evidence of this effect in the long waiting times (expected times exceeding an hour) between disposition and discharge for patients with ESI 3 and lower (see supplemental S1 Fig). Several strategies for reducing this delay in inpatient care have been reported in the literature, including development of a designated acute medical team to deliver full inpatient care while still residing at the ED [28] and a bed reservation policy that recommends bed reservation times for patients [31].

There are several limitations of our study that should be noted. For example, we discarded patient visits with seemingly invalid or inaccurate transition times, e.g. the discharge time occurred prior to first physician contact. Some of these transition sequences could be credible, while others are most likely data entry mistakes. While removal of these subjects could exhibit some bias on our results, we feel the impact is likely minimal and that our main conclusions remain valid. Another limitation is that our analysis did not include information on nurse staffing and number of laboratory tests which are relevant factors that could be included in further study [10, 32]. Also, the physical capacity of the ED (number of rooms) did not vary during this time and was not addressed in our model. Overall ED functional capacity is a complex issue which incorporates nursing and physician staffing, physical capacity of the ED, and the interplay with inpatient capacity of the hospital. Lastly, the Cox model assumes proportional hazards and while this assumption was met for most covariates, it was violated for acuity level particularly in the initial transition from registration to exam room. This could be addressed by incorporating time-dependent covariates, or through use of a more flexible modeling structure. In particular, Aalen’s generalized linear model is an alternative approach which permits flexible time-varying regression coefficients and confidence intervals and allows for non-proportional hazards [33].

In conclusion, reduction in ED waiting times is an important goal for emergency departments and understanding the factors which influence the overall ED process is an important first step towards achieving that goal. The multistate model presented in this paper decomposes the overall ED length of stay into constituent transitions and allows investigators to model covariate-specific effects on each transition. This allows physicians to identify which potentially modifiable covariates would have the greatest impact on the waiting times in each state in the model. Future research to expand on the multistate model with additional covariates and incorporate causal estimation will further improve our assessment and estimation of ED waiting times.

## Supporting information

S1 Data ED data for all subjects included in this study (de-identified). Data is made available as a comma-delimited file.
(CSV)

S2 Data Description of all variables included in the data set. (DOCX)

S1 Code R code to reproduce all results in the study. Code is made available as an R markdown.
(RMD)

S1 Table Cox models for all transitions in the multistate model. (DOCX)

S1 Fig Expected waiting time (sojourn time) in each state for different acuity levels. Waiting times are conditional on age = 1 year, gender = male, ethnicity = not Hispanic or Latino, race = white, time of the day = 16:00–20:00, season = winter, and number of ED physicians = 8.
(TIFF)

S2 Fig Expected waiting time (sojourn time) in each state for different seasons. Waiting times are conditional on age = 1 year, gender = male, ethnicity = not Hispanic or Latino, race = white, time of the day = 16:00–20:00, ESI = 4, and number of ED physicians = 8.
(TIF)

S3 Fig Expected waiting time (sojourn time) in each state for different times of day. Waiting times are conditional on age = 1 year, gender = male, ethnicity = not Hispanic or Latino, race = white, time of the day = 16:00–20:00, ESI = 4. The number of ED physicians for each time of day was set to the median value for that period, as follows: 0:00–4:00 = 7, 4:00–8:00 = 3, 8:00–12:00 = 5, 12:00–16:00 = 6, 16:00–20:00 = 8, 20:00–24:00 = 8.
(TIF)

S4 Fig Stacked transition probabilities of the ED multistate model for race. Transition probabilities are conditional on age = 1 year, gender = male, ethnicity = not Hispanic or Latino, ESI = 4, time of the day = 16:00–20:00, ESI = 4, and number of ED physicians = 8.
(TIF)

S5 Fig Expected waiting time (sojourn time) in each state for different races. Waiting times are conditional on age = 1 year, gender = male, ethnicity = not Hispanic or Latino, ESI = 4, time of the day = 16:00–20:00, ESI = 4, and number of ED physicians = 8.
(TIF)

S6 Fig Stacked transition probabilities of the ED multistate model for different ethnicity levels. Transition probabilities are conditional on age = 1 year, gender = male, race = white, ESI = 4, time of the day = 16:00–20:00, ESI = 4, and number of ED physicians = 8.
(TIF)

S7 Fig Expected waiting time (sojourn time) in each state for different acuity (ESI) levels. Waiting times are conditional on age = 1 year, gender = male, race = white, ESI = 4, time of the day = 16:00–20:00, ESI = 4, and number of ED physicians = 8.
(TIF)

S8 Fig Stacked transition probabilities of the ED multistate model for different acuity (ESI) levels. Transition probabilities are conditional on age = 1 year, race = white, ethnicity = not Hispanic or Latino, ESI = 4, time of the day = 16:00–20:00, ESI = 4, and number of ED physicians = 8.
(TIF)

S9 Fig Expected waiting time (sojourn time) in each state for different acuity (ESI) levels. Waiting times are conditional on age = 1 year, race = white, ethnicity = not Hispanic or Latino, ESI = 4, time of the day = 16:00–20:00, ESI = 4, and number of ED physicians = 8.
(TIF)

S10 Fig Plot of log(-log(S(t))) vs. log time for different ESI levels for each transition. Non-parallel curves indicate violation of the proportional hazards (PH) assumption. Note that ESI = 1 is not plotted on Reg -> Left and Reg -> Transfer panels, as no subjects with ESI = 1 were transferred or left without being seen.
(TIF)
