# Supplementary material for: Multistate model of the patient flow process in the pediatric emergency department
Source: PLoS One. 2019 Jul 10;14(7):e0219514. doi: 10.1371/journal.pone.0219514 (PMC6619791; doi:10.1371/journal.pone.0219514)
Supplement: S2 Data — (DOCX) [file pone.0219514.s002.docx]

**Variable descriptions for data associated with “Multistate model of the patient flow process in the pediatric emergency department”**

1. **First.Recorded.Acuity** – Initial Emergency Severity Index (ESI) level recorded at registration (1 = higher acuity / most urgent, 5 = lower acuity / least urgent)
2. **Acuity.Level** – ESI level recorded at time of triage, which could be different from initial recorded ESI
3. **Did.Acuity.Change** – Whether ESI changed between initial recording at registration and subsequent value at time of triage (YES/NO)
4. **Age** – Age of patient in years. Note that ages in the range 36-39, 40-44, 45-49, 50-54, 55-59, and 60+ were lumped together to avoid small cell counts, and patients were assigned the mid-point value of the age range.
5. **Ethnicity** – Ethnicity of patient, with possible values “Not Hispanic or Latino Unknown”, “Hispanic or Latino”, or “Other”
6. **Race** – Race of patient, with possible values “BLACK OR AFRICAN AMERICAN”, “WHITE”, “ASIAN”, “OTHER”, or “UNKNOWN”
7. **Gender** – Gender of patient, with possible values “M” (Male) and “F” (Female)
8. **Season** – Season of patient admission, with possible values “Spring”, “Summer”, “Fall”, “Winter”
9. **Time.of.day** – Time of day (in military time hours) for patient admission, with possible values “0-4h”, “4-8h”, “8-12h”, “12-16h”, “16-20h”, “20-24h”
10. **staff.count** – Number of physicians on staff at time of registration
11. **room** – Time from registration to exam room in hours
12. **contact** – Time from exam room to first contact with a physician, in hours
13. **disposition** – Time from first contact with a physician to disposition, in hours
14. **departure** – Time from disposition to discharge from ED, in hours
15. **triage** – Time from registration to triage, in hours
16. **left** – Time from registration to left without being seen, in hours
17. **redirect** – Time from registration to redirect within hospital, in hours
18. **room.s** – Exam room status (1 = observed, 0 = censored)
19. **contact.s** – Contact with physician status (1 = observed, 0 = censored)
20. **disposition.s** – Disposition status (1 = observed, 0 = censored)
21. **departure.s** – Departure status (1 = observed, 0 = censored)
22. **left.s** – Left without being seen status (1 = observed, 0 = censored)
23. **redirect.s** – Redirect within hospital status (1 = observed, 0 = censored)
