# Supplementary material for: Multistate model of the patient flow process in the pediatric emergency department
Source: PLoS One. 2019 Jul 10;14(7):e0219514. doi: 10.1371/journal.pone.0219514 (PMC6619791; doi:10.1371/journal.pone.0219514)
Supplement: S1 Table — (DOCX) [file pone.0219514.s004.docx]

**Table S1**. Cox models for all transitions in the multistate model

| **Transition** | **Variable** | **Levels** | **HR** | **95% CI** | **P-value** |
| --- | --- | --- | --- | --- | --- |
| **Reg->Room** | Age |  | 1.004 | 1.003, 1.005 | <0.001 |
|  | Gender | Male | Reference |  |  |
|  |  | Female | 0.963 | 0.949, 0.977 | <0.001 |
|  | Ethnicity | Not Hispanic | Reference |  |  |
|  |  | Hispanic | 1.029 | 0.994, 1.066 | 0.108 |
|  |  | Other | 0.97 | 0.908, 1.036 | 0.363 |
|  |  | Unknown | 0.95 | 0.861, 1.047 | 0.299 |
|  | Race | White | Reference |  |  |
|  |  | Black | 0.999 | 0.983, 1.016 | 0.94 |
|  |  | Asian | 1.029 | 0.982, 1.078 | 0.228 |
|  |  | Other | 0.99 | 0.962, 1.018 | 0.472 |
|  |  | Unknown | 0.957 | 0.919, 0.997 | 0.035 |
|  | Acuity (ESI) | 1 | Reference |  |  |
|  |  | 2 | 0.224 | 0.182, 0.276 | <0.001 |
|  |  | 3 | 0.09 | 0.073, 0.11 | <0.001 |
|  |  | 4 | 0.075 | 0.061, 0.092 | <0.001 |
|  |  | 5 | 0.069 | 0.056, 0.085 | <0.001 |
|  | Season | Spring | Reference |  |  |
|  |  | Summer | 1.426 | 1.395, 1.458 | <0.001 |
|  |  | Fall | 0.61 | 0.598, 0.623 | <0.001 |
|  |  | Winter | 0.58 | 0.568, 0.592 | <0.001 |
|  | Time of the day | 0:00 - 4:00 | Reference |  |  |
|  |  | 4:00 - 8:00 | 2.193 | 2.094, 2.296 | <0.001 |
|  |  | 8:00 - 12:00 | 3.056 | 2.959, 3.157 | <0.001 |
|  |  | 12:00 - 16:00 | 1.784 | 1.731, 1.838 | <0.001 |
|  |  | 16:00 - 20:00 | 1.874 | 1.813, 1.936 | <0.001 |
|  |  | 20:00 - 24:00 | 1.549 | 1.497, 1.602 | <0.001 |
|  | Number of ED physicians |  | 0.918 | 0.911, 0.925 | <0.001 |
| **Reg->Left** | Age |  | 0.998 | 0.991, 1.004 | 0.45 |
|  | Gender | Male | Reference |  |  |
|  |  | Female | 1.002 | 0.937, 1.071 | 0.962 |
|  | Ethnicity | Not Hispanic | Reference |  |  |
|  |  | Hispanic | 0.507 | 0.425, 0.604 | <0.001 |
|  |  | Other | 0.941 | 0.708, 1.249 | 0.672 |
|  |  | Unknown | 1.769 | 1.285, 2.433 | <0.001 |
|  | Race | White | Reference |  |  |
|  |  | Black | 1.13 | 1.047, 1.219 | 0.002 |
|  |  | Asian | 0.434 | 0.332, 0.567 | <0.001 |
|  |  | Other | 1.142 | 1.009, 1.293 | 0.036 |
|  |  | Unknown | 1.151 | 0.973, 1.361 | 0.1 |
|  | ESI | 1-2 | Reference |  |  |
|  |  | 3 | 1.716 | 1.214, 2.427 | 0.002 |
|  |  | 4 | 3.082 | 2.188, 4.343 | <0.001 |
|  |  | 5 | 3.702 | 2.62, 5.231 | <0.001 |
|  | Season | Spring | Reference |  |  |
|  |  | Summer | 1.223 | 1.076, 1.39 | 0.002 |
|  |  | Fall | 0.79 | 0.718, 0.869 | <0.001 |
|  |  | Winter | 0.715 | 0.65, 0.787 | <0.001 |
|  | Time of the day | 0:00 - 4:00 | Reference |  |  |
|  |  | 4:00 - 8:00 | 1.588 | 1.296, 1.946 | <0.001 |
|  |  | 8:00 - 12:00 | 1.204 | 1.015, 1.427 | 0.033 |
|  |  | 12:00 - 16:00 | 0.965 | 0.861, 1.082 | 0.546 |
|  |  | 16:00 - 20:00 | 1.085 | 0.961, 1.225 | 0.187 |
|  |  | 20:00 - 24:00 | 0.996 | 0.893, 1.11 | 0.937 |
|  | Number of ED physicians |  | 0.952 | 0.925, 0.98 | 0.001 |
| **Reg->Transfer** | Age |  | 1.028 | 1.014, 1.042 | <0.001 |
|  | Gender | Male | Reference |  |  |
|  |  | Female | 1.263 | 1.092, 1.46 | 0.002 |
|  | Ethnicity | Not Hispanic | Reference |  |  |
|  |  | Hispanic | 0.812 | 0.586, 1.126 | 0.212 |
|  |  | Other | 0.903 | 0.488, 1.67 | 0.745 |
|  |  | Unknown | 1.353 | 0.596, 3.069 | 0.47 |
|  | Race | White | Reference |  |  |
|  |  | Black | 1.091 | 0.925, 1.288 | 0.299 |
|  |  | Asian | 0.821 | 0.501, 1.346 | 0.435 |
|  |  | Other | 1.161 | 0.886, 1.521 | 0.279 |
|  |  | Unknown | 0.915 | 0.626, 1.337 | 0.645 |
|  | ESI | 1-2 | Reference |  |  |
|  |  | 3 | 0.06 | 0.037, 0.099 | <0.001 |
|  |  | 4 | 0.483 | 0.359, 0.651 | <0.001 |
|  |  | 5 | 2.981 | 2.256, 3.938 | <0.001 |
|  | Season | Spring | Reference |  |  |
|  |  | Summer | 2.674 | 2.131, 3.355 | <0.001 |
|  |  | Fall | 0.743 | 0.594, 0.928 | 0.009 |
|  |  | Winter | 1.065 | 0.865, 1.312 | 0.553 |
|  | Time of the day | 0:00 - 4:00 | Reference |  |  |
|  |  | 4:00 - 8:00 | 3.248 | 1.593, 6.621 | 0.001 |
|  |  | 8:00 - 12:00 | 13.439 | 7.821, 23.092 | <0.001 |
|  |  | 12:00 - 16:00 | 16.555 | 9.929, 27.603 | <0.001 |
|  |  | 16:00 - 20:00 | 25.091 | 14.762, 42.646 | <0.001 |
|  |  | 20:00 - 24:00 | 10.276 | 5.928, 17.811 | <0.001 |
|  | Number of ED physicians |  | 0.848 | 0.786, 0.914 | <0.001 |
| **Room->Contact** | Age |  | 1.003 | 1.002, 1.004 | <0.001 |
|  | Gender | Male | Reference |  |  |
|  |  | Female | 0.962 | 0.948, 0.977 | <0.001 |
|  | Ethnicity | Not Hispanic | Reference |  |  |
|  |  | Hispanic | 0.959 | 0.926, 0.993 | 0.02 |
|  |  | Other | 0.971 | 0.909, 1.038 | 0.391 |
|  |  | Unknown | 1.115 | 1.012, 1.229 | 0.028 |
|  | Race | White | Reference |  |  |
|  |  | Black | 0.987 | 0.971, 1.003 | 0.12 |
|  |  | Asian | 0.901 | 0.86, 0.944 | <0.001 |
|  |  | Other | 1.025 | 0.996, 1.055 | 0.085 |
|  |  | Unknown | 0.974 | 0.935, 1.015 | 0.21 |
|  | Acuity (ESI) | 1 | Reference |  |  |
|  |  | 2 | 0.916 | 0.752, 1.115 | 0.382 |
|  |  | 3 | 0.559 | 0.459, 0.68 | <0.001 |
|  |  | 4 | 0.497 | 0.408, 0.605 | <0.001 |
|  |  | 5 | 0.491 | 0.403, 0.598 | <0.001 |
|  | Season | Spring | Reference |  |  |
|  |  | Summer | 1.18 | 1.155, 1.206 | <0.001 |
|  |  | Fall | 0.94 | 0.92, 0.959 | <0.001 |
|  |  | Winter | 0.945 | 0.926, 0.964 | <0.001 |
|  | Time of the day | 0:00 - 4:00 | Reference |  |  |
|  |  | 4:00 - 8:00 | 1.239 | 1.184, 1.297 | <0.001 |
|  |  | 8:00 - 12:00 | 1.723 | 1.669, 1.779 | <0.001 |
|  |  | 12:00 - 16:00 | 1.52 | 1.475, 1.565 | <0.001 |
|  |  | 16:00 - 20:00 | 1.653 | 1.601, 1.707 | <0.001 |
|  |  | 20:00 - 24:00 | 1.34 | 1.295, 1.386 | <0.001 |
|  | Number of ED physicians |  | 0.999 | 0.992, 1.006 | 0.754 |
| **Contact->Disp** | Age |  | 0.99 | 0.989, 0.991 | <0.001 |
|  | Gender | Male | Reference |  |  |
|  |  | Female | 0.935 | 0.921, 0.949 | <0.001 |
|  | Ethnicity | Not Hispanic | Reference |  |  |
|  |  | Hispanic | 0.995 | 0.961, 1.031 | 0.8 |
|  |  | Other | 1.052 | 0.985, 1.124 | 0.134 |
|  |  | Unknown | 1.122 | 1.018, 1.236 | 0.02 |
|  | Race | White | Reference |  |  |
|  |  | Black | 1.042 | 1.025, 1.06 | <0.001 |
|  |  | Asian | 0.963 | 0.919, 1.009 | 0.111 |
|  |  | Other | 1.056 | 1.026, 1.087 | <0.001 |
|  |  | Unknown | 0.958 | 0.919, 0.998 | 0.038 |
|  | Acuity (ESI) | 1 | Reference |  |  |
|  |  | 2 | 0.622 | 0.511, 0.757 | <0.001 |
|  |  | 3 | 0.702 | 0.577, 0.855 | <0.001 |
|  |  | 4 | 2.562 | 2.105, 3.119 | <0.001 |
|  |  | 5 | 3.942 | 3.236, 4.802 | <0.001 |
|  | Season | Spring | Reference |  |  |
|  |  | Summer | 0.925 | 0.905, 0.945 | <0.001 |
|  |  | Fall | 0.93 | 0.911, 0.949 | <0.001 |
|  |  | Winter | 0.924 | 0.905, 0.943 | <0.001 |
|  | Time of the day | 0:00 - 4:00 | Reference |  |  |
|  |  | 4:00 - 8:00 | 0.918 | 0.877, 0.961 | <0.001 |
|  |  | 8:00 - 12:00 | 0.835 | 0.809, 0.862 | <0.001 |
|  |  | 12:00 - 16:00 | 0.829 | 0.805, 0.854 | <0.001 |
|  |  | 16:00 - 20:00 | 0.857 | 0.83, 0.885 | <0.001 |
|  |  | 20:00 - 24:00 | 1.018 | 0.985, 1.053 | 0.29 |
|  | Number of ED physicians |  | 1.019 | 1.012, 1.027 | <0.001 |
| **Disp->Depart** | Age |  | 1.002 | 1.001, 1.004 | <0.001 |
|  | Gender | Male | Reference |  |  |
|  |  | Female | 1.018 | 1.003, 1.033 | 0.02 |
|  | Ethnicity | Not Hispanic | Reference |  |  |
|  |  | Hispanic | 0.943 | 0.91, 0.977 | 0.001 |
|  |  | Other | 1.041 | 0.975, 1.112 | 0.23 |
|  |  | Unknown | 1.045 | 0.948, 1.152 | 0.371 |
|  | Race | White | Reference |  |  |
|  |  | Black | 1.016 | 0.999, 1.033 | 0.06 |
|  |  | Asian | 0.939 | 0.896, 0.983 | 0.007 |
|  |  | Other | 1.025 | 0.996, 1.055 | 0.088 |
|  |  | Unknown | 1.004 | 0.964, 1.047 | 0.832 |
|  | Acuity (ESI) | 1 | Reference |  |  |
|  |  | 2 | 0.923 | 0.758, 1.124 | 0.425 |
|  |  | 3 | 1.421 | 1.167, 1.729 | <0.001 |
|  |  | 4 | 2.149 | 1.766, 2.616 | <0.001 |
|  |  | 5 | 2.775 | 2.278, 3.38 | <0.001 |
|  | Season | Spring | Reference |  |  |
|  |  | Summer | 1.001 | 0.98, 1.023 | 0.916 |
|  |  | Fall | 0.972 | 0.952, 0.992 | 0.007 |
|  |  | Winter | 0.96 | 0.94, 0.979 | <0.001 |
|  | Time of the day | 0:00 - 4:00 | Reference |  |  |
|  |  | 4:00 - 8:00 | 0.948 | 0.905, 0.991 | 0.02 |
|  |  | 8:00 - 12:00 | 0.886 | 0.858, 0.914 | <0.001 |
|  |  | 12:00 - 16:00 | 0.897 | 0.871, 0.924 | <0.001 |
|  |  | 16:00 - 20:00 | 0.906 | 0.878, 0.936 | <0.001 |
|  |  | 20:00 - 24:00 | 0.946 | 0.915, 0.978 | 0.001 |
|  | Number of ED physicians |  | 1.002 | 0.995, 1.009 | 0.577 |
