# Supplementary figures and images for: Multistate model of the patient flow process in the pediatric emergency department
Source: PLoS One. 2019 Jul 10;14(7):e0219514. doi: 10.1371/journal.pone.0219514 (PMC6619791; doi:10.1371/journal.pone.0219514)

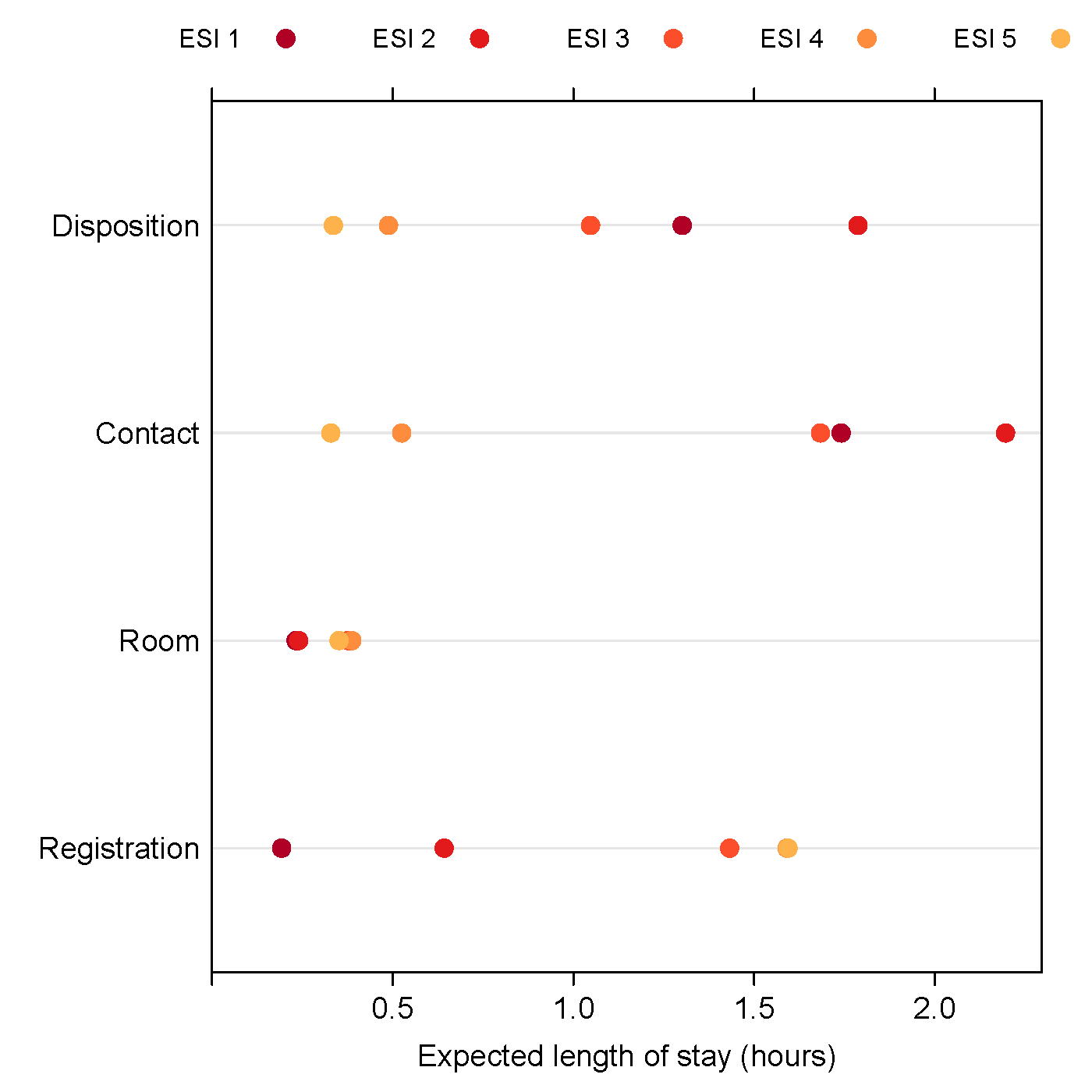

Supplement: S1 Fig — Waiting times are conditional on age = 1 year, gender = male, ethnicity = not Hispanic or Latino, race = white, time of the day = 16:00–20:00, season = winter, and number of ED physicians = 8. (TIFF) [file pone.0219514.s005.tiff]

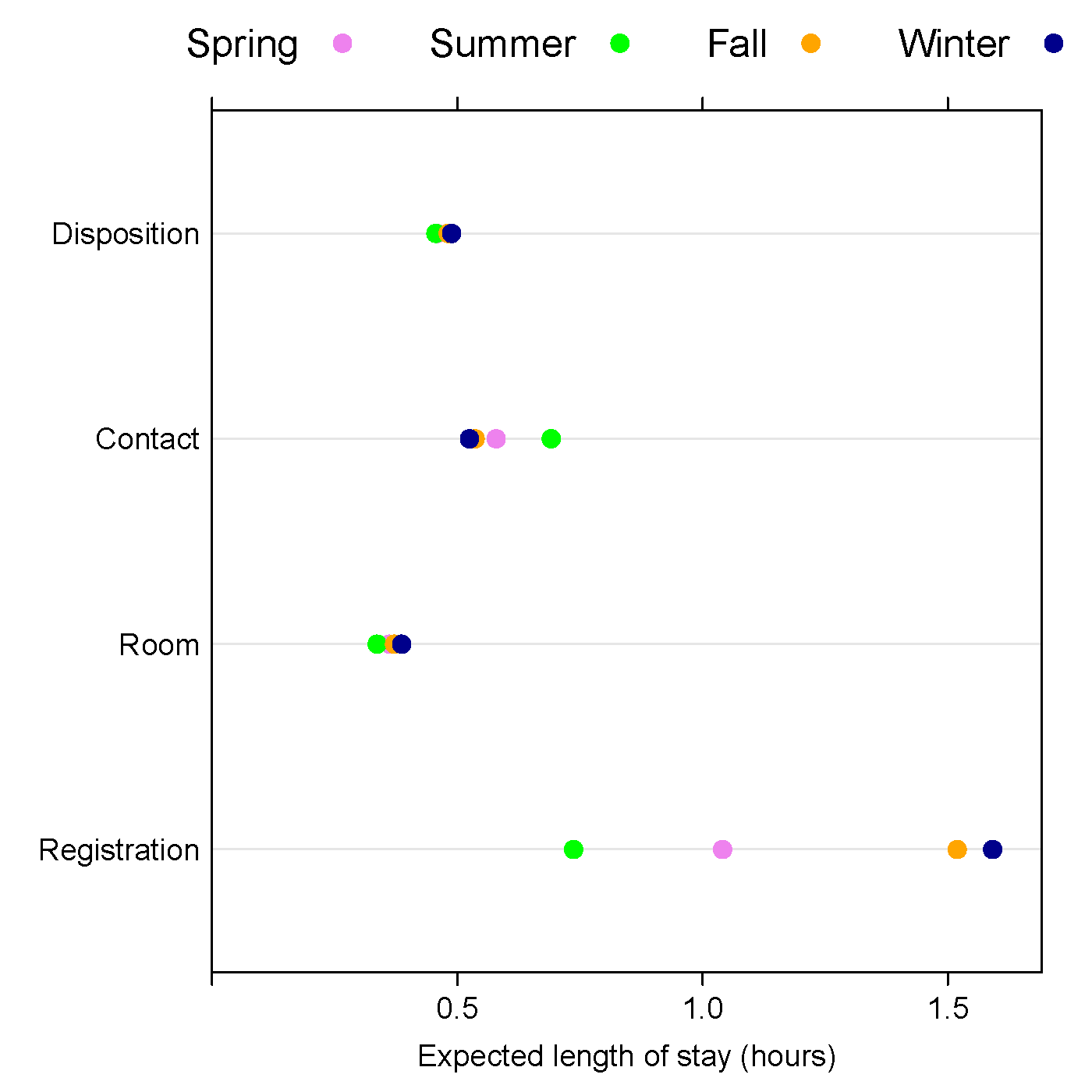

Supplement: S2 Fig — Waiting times are conditional on age = 1 year, gender = male, ethnicity = not Hispanic or Latino, race = white, time of the day = 16:00–20:00, ESI = 4, and number of ED physicians = 8. (TIF) [file pone.0219514.s006.tif]

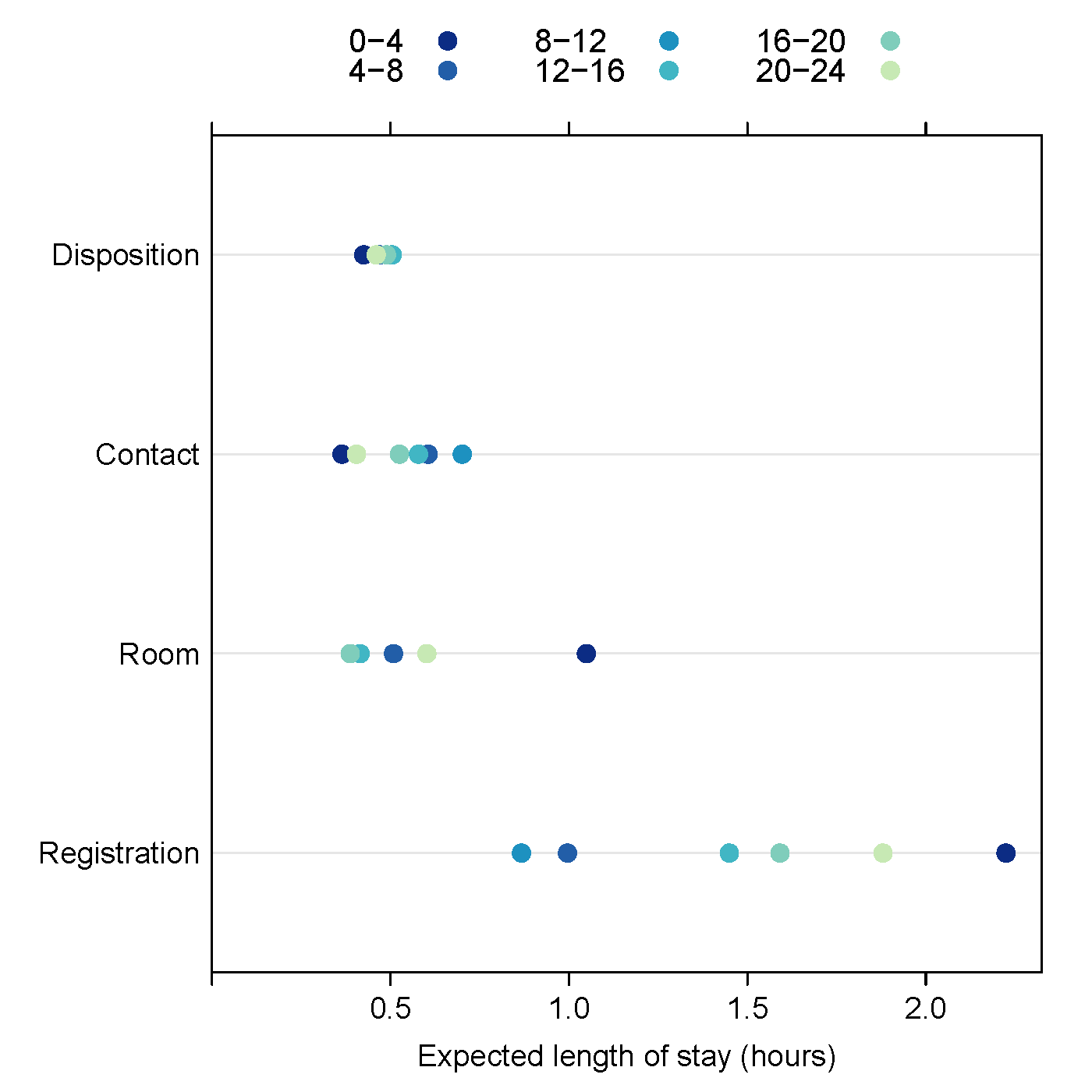

Supplement: S3 Fig — Waiting times are conditional on age = 1 year, gender = male, ethnicity = not Hispanic or Latino, race = white, time of the day = 16:00–20:00, ESI = 4. The number of ED physicians for each time of day was set to the median value for that period, as follows: 0:00–4:00 = 7, 4:00–8:00 = 3, 8:00–12:00 = 5, 12:00–16:00 = 6, 16:00–20:00 = 8, 20:00–24:00 = 8. (TIF) [file pone.0219514.s007.tif]

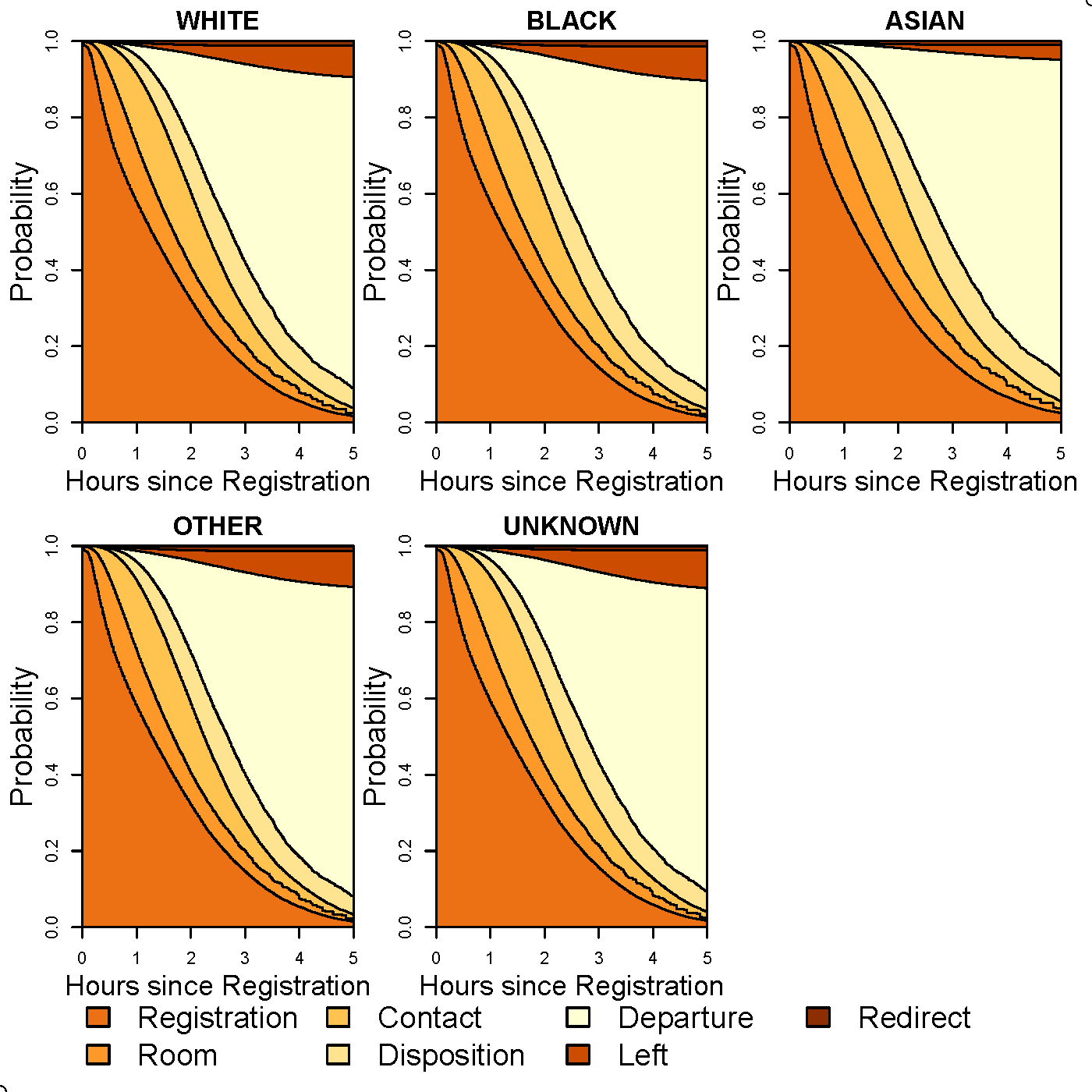

Supplement: S4 Fig — Transition probabilities are conditional on age = 1 year, gender = male, ethnicity = not Hispanic or Latino, ESI = 4, time of the day = 16:00–20:00, ESI = 4, and number of ED physicians = 8. (TIF) [file pone.0219514.s008.tif]

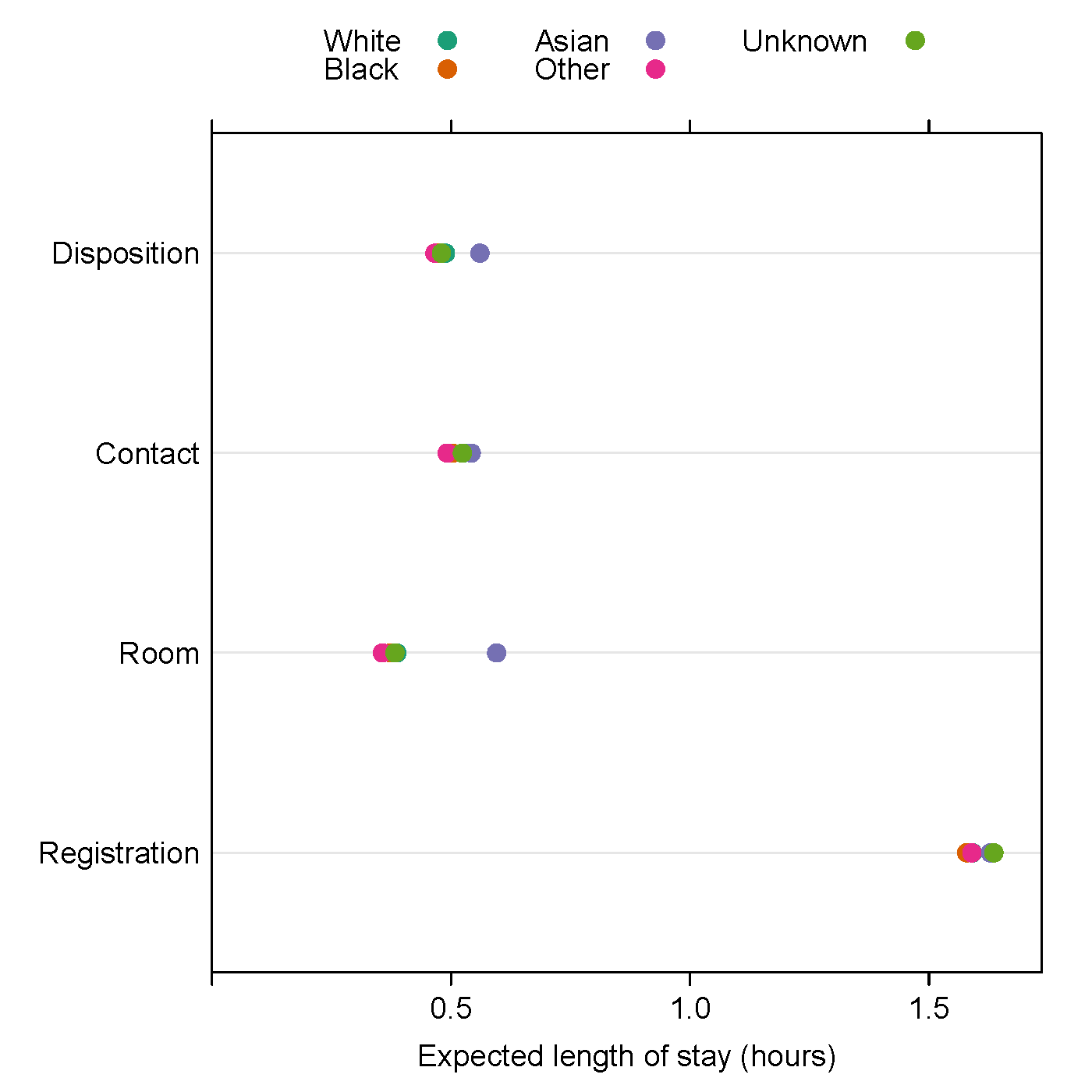

Supplement: S5 Fig — Waiting times are conditional on age = 1 year, gender = male, ethnicity = not Hispanic or Latino, ESI = 4, time of the day = 16:00–20:00, ESI = 4, and number of ED physicians = 8. (TIF) [file pone.0219514.s009.tif]

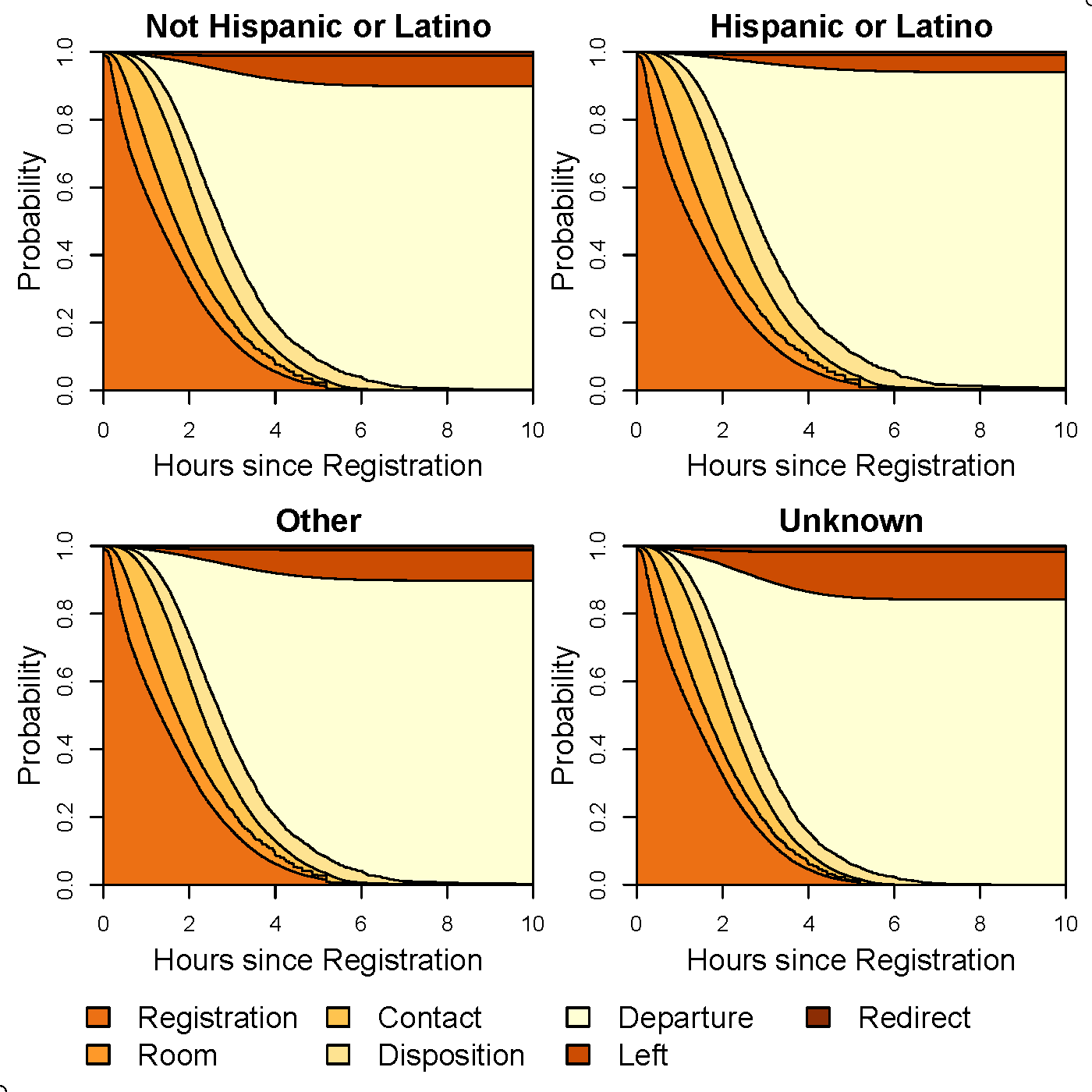

Supplement: S6 Fig — Transition probabilities are conditional on age = 1 year, gender = male, race = white, ESI = 4, time of the day = 16:00–20:00, ESI = 4, and number of ED physicians = 8. (TIF) [file pone.0219514.s010.tif]

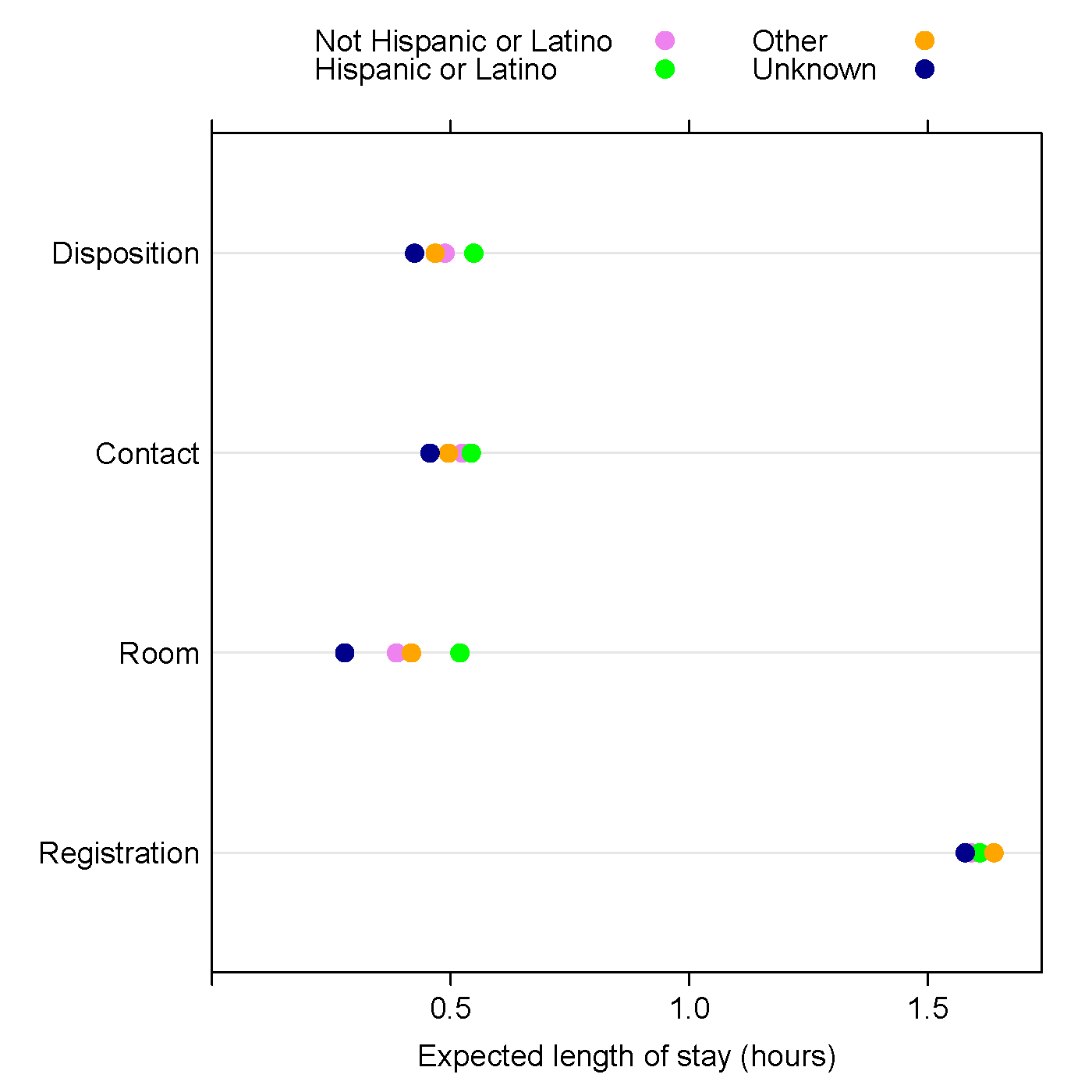

Supplement: S7 Fig — Waiting times are conditional on age = 1 year, gender = male, race = white, ESI = 4, time of the day = 16:00–20:00, ESI = 4, and number of ED physicians = 8. (TIF) [file pone.0219514.s011.tif]

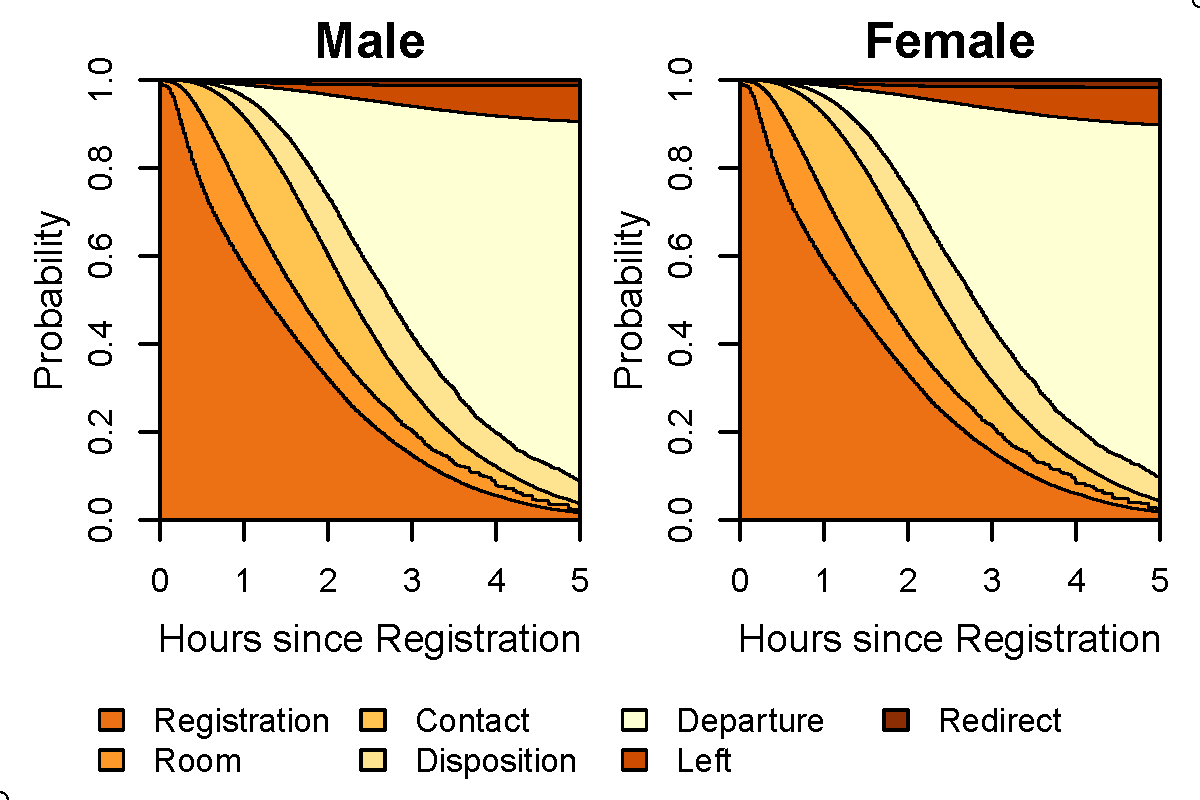

Supplement: S8 Fig — Transition probabilities are conditional on age = 1 year, race = white, ethnicity = not Hispanic or Latino, ESI = 4, time of the day = 16:00–20:00, ESI = 4, and number of ED physicians = 8. (TIF) [file pone.0219514.s012.tif]

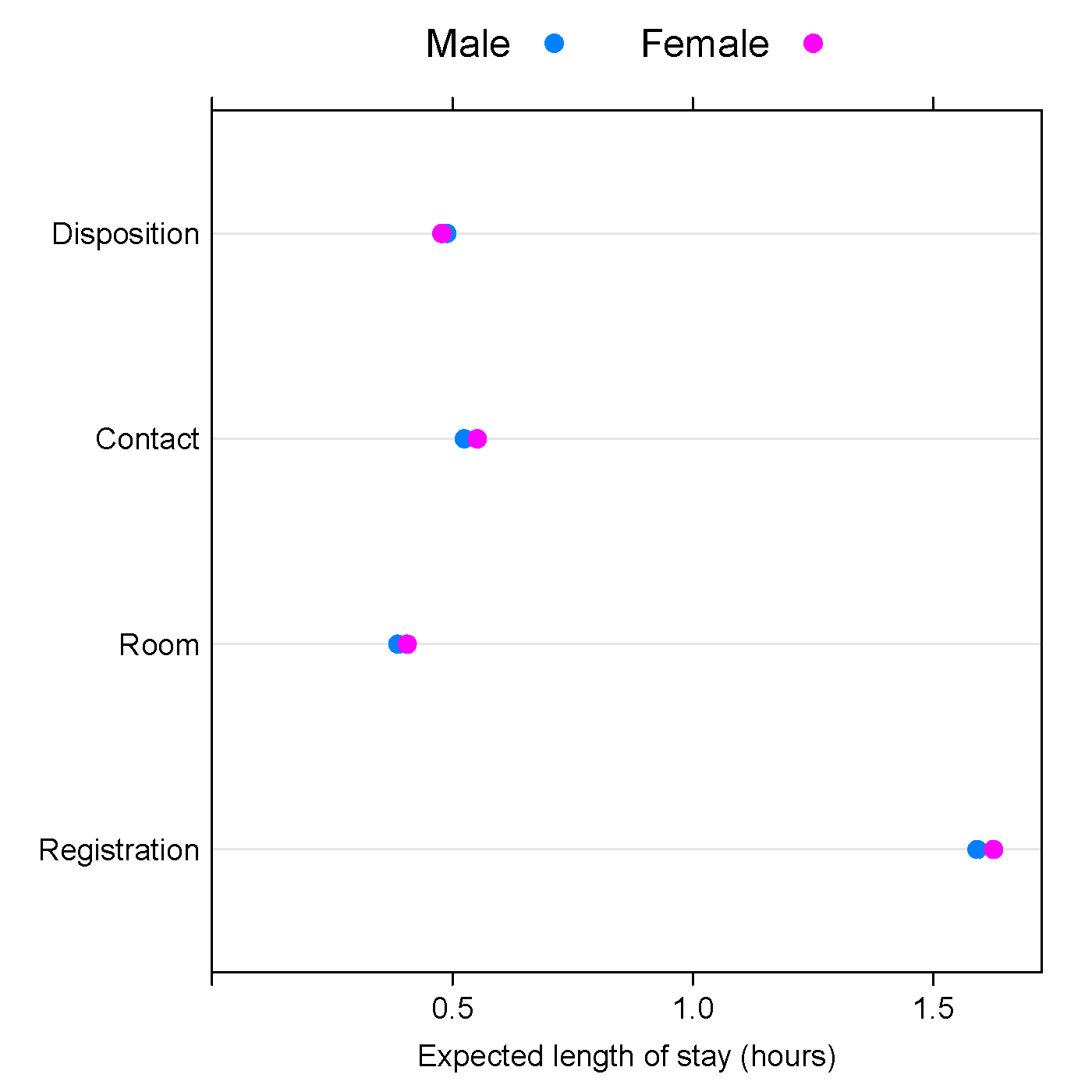

Supplement: S9 Fig — Waiting times are conditional on age = 1 year, race = white, ethnicity = not Hispanic or Latino, ESI = 4, time of the day = 16:00–20:00, ESI = 4, and number of ED physicians = 8. (TIF) [file pone.0219514.s013.tif]

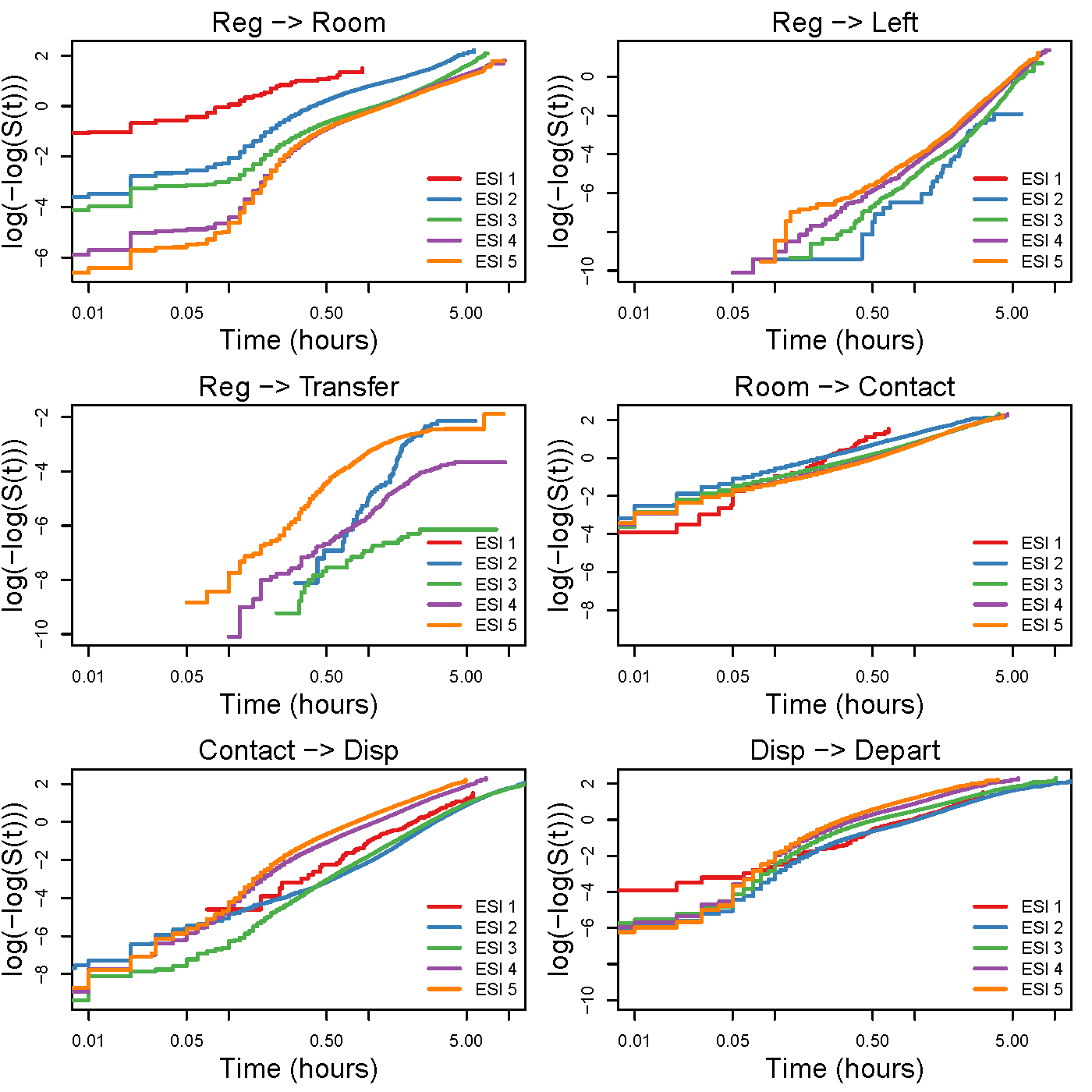

Supplement: S10 Fig — Non-parallel curves indicate violation of the proportional hazards (PH) assumption. Note that ESI = 1 is not plotted on Reg -> Left and Reg -> Transfer panels, as no subjects with ESI = 1 were transferred or left without being seen. (TIF) [file pone.0219514.s014.tif]
